# A stepwise fresh porcine head model for three-dimensional dissection of ventricular, limbic, and deep venous anatomy

**DOI:** 10.1371/journal.pone.0361913

**Published:** 2026-11-06

**Authors:** Sara Ganaha, Abdi Ermolo, Yuji Katayama, Yohei Kitamura

**Affiliations:** 1 Department of Neurosurgery, Keio University School of Medicine, Tokyo, Japan; 2 Department of Neurosurgery, St. Peter's Specialized Hospital, Addis Ababa, Ethiopia; 3 Department of Ophthalmology, National Hospital Organization Shizuoka Medical Center, Shizuoka, Japan; Namik Kemal University: Tekirdag Namik Kemal Universitesi, TÜRKIYE

## Abstract

Deep ventricular, limbic, cisternal, and deep venous structures form a compact three-dimensional anatomical system that is difficult to understand from two-dimensional resources alone. We evaluated the technical feasibility of organizing fresh *ex vivo* porcine heads into a four-module, stepwise dissection model for demonstrating these relationships. Five porcine heads obtained from a regulated food supply chain were prepared without formalin fixation, vascular injection, or specialized laboratory equipment. A neurosurgery resident performed the dissections under faculty supervision. Anatomical feasibility was assessed by the successful identification of prespecified targets within transcallosal, transchoroidal, interforniceal, posterior deep venous, and atrial-inferior horn dissections. The complete four-module sequence was achieved, and all prespecified anatomical targets were identified during dissection in all five specimens. In an independent image-based assessment of three specimens with complete unannotated photographic sets, 26 of 31 targets were identifiable in all three specimens, whereas five deep ventricular or venous targets were identifiable in two of three specimens. The dissection demonstrated the spatial continuity of the caudate nucleus, fornix, foramen of Monro, choroid plexus, thalamostriate vein, velum interpositum, internal cerebral veins, Galenic venous system, hippocampal formation, choroidal fissure, and adjacent cisternal spaces. Natural vascular coloration remained visible without injection and supported orientation during sequential dissection. This low-infrastructure preparation may serve as an accessible physical reference for understanding the three-dimensional continuity of deep ventricular, limbic, cisternal, and venous anatomy, particularly where access to human cadaveric dissection is limited.

## Introduction

Human cadaveric dissection remains the reference standard for learning neurosurgical anatomy because it permits direct appreciation of tissue planes, operative corridors, and three-dimensional relationships. Access, however, is restricted in many institutions by specimen availability, cost, regulation, and the need for dedicated facilities. Large-animal preparations have therefore been explored as complementary resources for anatomical teaching and selected procedural simulation, particularly in settings where human cadaver laboratories are not readily available [1–4]. Porcine specimens are attractive for this purpose because the brain is sufficiently large for microsurgical manipulation, fresh tissue planes can be preserved, and pig heads can be obtained as low-value food-industry by-products that might otherwise be underutilized or discarded, without requiring purpose-bred animal experimentation.

The ventricular system, limbic structures, choroidal fissure, basal cisterns, and deep cerebral veins are especially difficult to learn as an integrated anatomical system. These structures are compact, curved, and continuous around the thalamus and upper brainstem. Although textbooks, clinical imaging, and digital three-dimensional models are essential, they do not provide the same physical experience of tracing the fornix-hippocampal complex, opening the choroidal fissure, or following the internal cerebral veins toward the vein of Galen [5–12].

We previously described a low-cost fresh porcine head preparation for introductory teaching of selected cranial approaches and gross neuroanatomy [13]. The specimens used in the present study were independent of those used in our previous study. The present study addresses a distinct question: whether the same accessible preparation can support a structured, continuous dissection of deep ventricular, limbic, cisternal, and venous relationships. We therefore developed a four-module dissection sequence and evaluated its anatomical feasibility across five specimens. The study was designed to characterize the model and the consistency of anatomical visualization across specimens, not to measure learner performance or surgical competency.

## Materials and methods

### Study design and objectives

This descriptive anatomical feasibility study used five fresh *ex vivo* porcine heads to evaluate a standardized four-module dissection sequence focused on deep ventricular, limbic, choroidal fissure, cisternal, and venous anatomy. The primary outcome was successful completion of the entire four-module sequence in each specimen. Secondary outcomes were successful identification of the anatomical targets assigned to each module, according to the operational definitions presented in Table 1. The study was designed to characterize the technical feasibility and consistency of anatomical visualization across specimens rather than to evaluate learner performance, technical competency, or transfer of skills to human surgery.

**Table 1:** Anatomical target identification during dissection and independent image-based assessment across the four-module sequence.

	Anatomical target	Operational definition	Identification during dissection, n/N (%)	Independent image-based identification, n/N (%)
Module 1	Corpus callosum	Pale commissural structure identified beneath the medial hemispheric surface	5/5 (100%)	3/3 (100%)
	Lateral ventricle	Ventricular cavity entered through a limited midline callosotomy	5/5 (100%)	3/3 (100%)
	Caudate nucleus	Caudate structure identified adjacent to the lateral ventricle	5/5 (100%)	3/3 (100%)
	Fornix	Forniceal body/commissure identified in relation to the ventricle and thalamus	5/5 (100%)	3/3 (100%)
	Foramen of Monro	Communication between the lateral and third ventricular regions identified	5/5 (100%)	3/3 (100%)
	Choroid plexus	Choroid plexus identified within the lateral ventricle	5/5 (100%)	3/3 (100%)
	Thalamostriate vein	Thalamostriate vein identified in relation to the choroid plexus	5/5 (100%)	3/3 (100%)
	Thalamic surface	Thalamic surface exposed after medial mobilization of the fornix/choroid plexus	5/5 (100%)	3/3 (100%)
	Internal cerebral veins	Paired deep veins identified ventral to the fornix	5/5 (100%)	2/3 (66.7%)
	Third ventricle	Third ventricular region accessed or clearly demonstrated	5/5 (100%)	2/3 (66.7%)
Module 2	Choroidal fissure	Choroidal fissure opened near the foramen of Monro	5/5 (100%)	3/3 (100%)
	Anterior caudate vein	Venous branch identified converging toward the thalamostriate vein	5/5 (100%)	2/3 (66.7%)
	Posterior caudate vein	Venous branch identified converging toward the thalamostriate vein	5/5 (100%)	2/3 (66.7%)
	Anterior septal vein	Venous branch identified near the foramen of Monro	5/5 (100%)	2/3 (66.7%)
	Velum interpositum	Velum interpositum exposed by following the thalamostriate vein medially	5/5 (100%)	3/3 (100%)
	Paired internal cerebral veins	Both internal cerebral veins identified beneath the fornix	5/5 (100%)	3/3 (100%)
Module 3	Upper brainstem/tectal region	Posterior upper brainstem and tectal region exposed	5/5 (100%)	3/3 (100%)
	Thalami	Thalami identified above the upper brainstem	5/5 (100%)	3/3 (100%)
	Fornix overlying thalami	Fornix demonstrated in relation to the thalami	5/5 (100%)	3/3 (100%)
	Posterior course of internal cerebral veins	Internal cerebral veins traced posteriorly	5/5 (100%)	3/3 (100%)
	Vein of Galen	Galenic venous confluence identified	5/5 (100%)	3/3 (100%)
	Straight sinus	Vein of Galen traced to the straight sinus/falcotentorial junction	5/5 (100%)	3/3 (100%)
Module 4	Atrium	Atrium of the lateral ventricle exposed	5/5 (100%)	3/3 (100%)
	Inferior horn	Inferior horn of the lateral ventricle exposed	5/5 (100%)	3/3 (100%)
	Fornix–hippocampal continuity	Continuous curved relationship between the fornix and hippocampal formation demonstrated	5/5 (100%)	3/3 (100%)
	Choroid plexus to inferior choroidal point	Choroid plexus traced to the inferior choroidal point	5/5 (100%)	3/3 (100%)
	Fimbria	Fimbria exposed after medial mobilization of the choroid plexus	5/5 (100%)	3/3 (100%)
	Choroidal fissure in inferior horn	Choroidal fissure identified/opened in the inferior horn	5/5 (100%)	3/3 (100%)
	Ambient cistern	Ambient cistern entered after opening the choroidal fissure	5/5 (100%)	3/3 (100%)
	Posterior cerebral arterial branches	Arterial branches identified along the brainstem surface	5/5 (100%)	3/3 (100%)
	Hippocampal head	Hippocampal head demonstrated within the inferior horn	5/5 (100%)	3/3 (100%)

Note. Independent image-based assessment was performed in the three specimens for which complete unannotated photographic sets covering all prespecified anatomical targets were available.

### Specimens and ethical review

Five fresh heads of domestic pigs (*Sus scrofa domesticus*) were obtained from a regulated food supply chain. Information regarding breed, sex, age, and the exact interval between slaughter and dissection was not available because the specimens were obtained as food-industry by-products. The protocol was reviewed by the Animal Laboratory Center at Keio University and was determined to be exempt from formal animal ethics approval because only nonliving specimens were used. Magnetic resonance images of one author, acquired during a routine health examination, were used solely as anatomical references and were not included in the analysis. The Ethics Committee of Keio University School of Medicine determined that this use did not constitute research involving human participants and therefore did not require formal ethical review (reference no. 26–084).

### Specimen preparation and dissection environment

The heads were prepared using the previously reported protocol [13]. The specimens were not formalin-fixed and did not undergo arterial or venous injection of dye. After removal of the skin, mandible, and soft tissues as needed, the calvarium and dura were removed to expose the cerebral hemispheres. Standard anatomical instruments, a manual saw, simple retractors, and personal protective equipment were used. The dissections were performed by a neurosurgery resident under the supervision of an attending neurosurgeon experienced in cerebrovascular and brain tumor surgery. Anatomical structures were reviewed during and after dissection, and photographs were obtained with a commercially available smartphone.

The specimens were stored at 4 °C until dissection and dissected at room temperature within 24 hours of receipt from the regulated food supplier. All five specimens underwent the complete four-module dissection sequence.

### Four-module dissection sequence and anatomical targets

The four modules were performed sequentially in all five specimens and were designed to demonstrate the three-dimensional continuity of the ventricular system, fornix–hippocampal complex, choroidal fissure, deep cerebral veins, and adjacent cisternal spaces.

#### Module 1: Interhemispheric, transcallosal, subchoroidal, and interforniceal dissection.

The interhemispheric fissure was opened along the arachnoid plane, and the corpus callosum was identified beneath the medial hemispheric surfaces. A limited midline callosotomy was performed to enter the lateral ventricle. The caudate nucleus, fornix, foramen of Monro, choroid plexus, and thalamostriate vein were identified. The fornix and choroid plexus were gently retracted medially to expose the thalamic surface and demonstrate the subchoroidal trajectory toward the third ventricle. The contralateral lateral ventricle was subsequently entered, and the internal cerebral veins were identified on the ventral surface of the fornix. The fornix was then divided at the midline to demonstrate the interforniceal route toward the third ventricle.

#### Module 2: Transchoroidal exposure of intraventricular veins.

Using the transcallosal ventricular entry established in Module 1, the choroidal fissure was opened near the foramen of Monro. The choroid plexus and thalamostriate vein were gently mobilized laterally to expose the underlying thalamic surface. The anterior caudate vein, posterior caudate vein, and anterior septal vein were identified in relation to the thalamostriate vein. The thalamostriate vein was then followed medially into the velum interpositum, where the paired internal cerebral veins were exposed beneath the fornix.

#### Module 3: Posterior exploration of the deep venous system.

The internal cerebral veins were followed posteriorly toward the Galenic venous confluence. The occipital lobes were elevated superiorly to expose the posterior upper brainstem, tectal region, and quadrigeminal cistern. The thalami and the broad fornix overlying their dorsal surfaces were identified. The internal cerebral veins were traced along the ventral surface of the fornix to the vein of Galen, which was subsequently followed toward the straight sinus at the falcotentorial junction.

#### Module 4: Atrium, inferior horn, limbic structures, and adjacent cisterns.

The lateral ventricular dissection was extended posteriorly into the atrium and laterally into the inferior horn. The upper portion of the cerebral cortex was removed by an axial cut immediately above the zygomatic root to provide a panoramic view of the lateral ventricle. The continuous curved relationship between the fornix and hippocampal formation was examined in relation to the thalamus. The choroid plexus was followed to the inferior choroidal point, and medial mobilization of the choroid plexus exposed the fimbria and choroidal fissure. The choroidal fissure was opened to enter the ambient cistern and identify adjacent posterior cerebral arterial branches. The hippocampal head was examined from within the inferior horn.

### Assessment of anatomical feasibility

The anatomical targets listed in Table 1 were documented during each dissection using contemporaneous photographs and dissection notes. Target identification was subsequently confirmed by review of these original records. A target was considered successfully identified when the structure and its relevant relationship to adjacent anatomical landmarks could be documented according to the operational definition in Table 1.

Venous tributaries were considered identified only when they were directly visualized as distinct vessels and their relationships with adjacent ventricular venous landmarks could be documented; vessel identity was not assigned solely on the basis of the expected human anatomical pattern.

To provide an independent assessment of image-based identifiability, an assessor who had not participated in the dissections or image acquisition reviewed the original unannotated photographs. The assessor was blinded to the investigators’ annotations and evaluated each prespecified anatomical target using the anatomical criteria corresponding to the operational definitions shown in Table 1. Each target was recorded as identifiable or not identifiable from the available photographs. Complete unannotated photographic sets covering all prespecified anatomical targets were available for three of the five specimens.

Completion of each module was defined as completion of all planned dissection steps for that module. Completion of the overall sequence required completion of all four modules in the same specimen. Results were recorded at the specimen level and are presented as n/N and percentage. No inferential statistical testing was performed because the study was descriptive and involved five specimens.

### Illustrative human magnetic resonance images

De-identified human brain magnetic resonance images were used only as illustrative qualitative references in Fig 4 and S3 Fig and were not included in the anatomical feasibility analysis. Written informed consent for publication of the images was obtained.

### Post-dissection handling and disposal

Post-dissection handling and disposal followed institutional procedures and applicable Japanese regulations for food-derived biological materials used for educational or research purposes. These procedures reflect local institutional practice and should be adapted to the requirements of each jurisdiction.

## Results

### Overall feasibility of the stepwise sequence

Removal of the calvarium and dura provided sufficient exposure for sequential examination of the deep ventricular, limbic, cisternal, and venous structures (S1 Fig). The fresh, non-fixed specimens retained recognizable tissue planes and natural vascular coloration, which supported anatomical orientation during the dissections.

The four-module dissection sequence was completed in all five specimens (5/5, 100%). All anatomical targets listed in Table 1 were assessable and successfully identified during dissection in all five specimens. Across the independent image-based assessment, 88 of 93 target–specimen observations (94.6%) were identifiable. Twenty-six of the 31 prespecified targets were identifiable in all three evaluable specimens, whereas five targets—the internal cerebral veins, third ventricle, anterior caudate vein, posterior caudate vein, and anterior septal vein—were identifiable in two of the three specimens. The target-specific results are shown in Table 1, and the underlying specimen-level data are provided in S1 Data.

### Module 1: Interhemispheric, transcallosal, subchoroidal, and interforniceal dissection

The corpus callosum and lateral ventricle were identified in all five specimens. The caudate nucleus, fornix, foramen of Monro, choroid plexus, and thalamostriate vein were clearly demonstrated within the lateral ventricle (Fig 1A–E). The spatial relationship between the thalamostriate vein and choroid plexus permitted determination of the side of ventricular entry.

**Fig 1:**
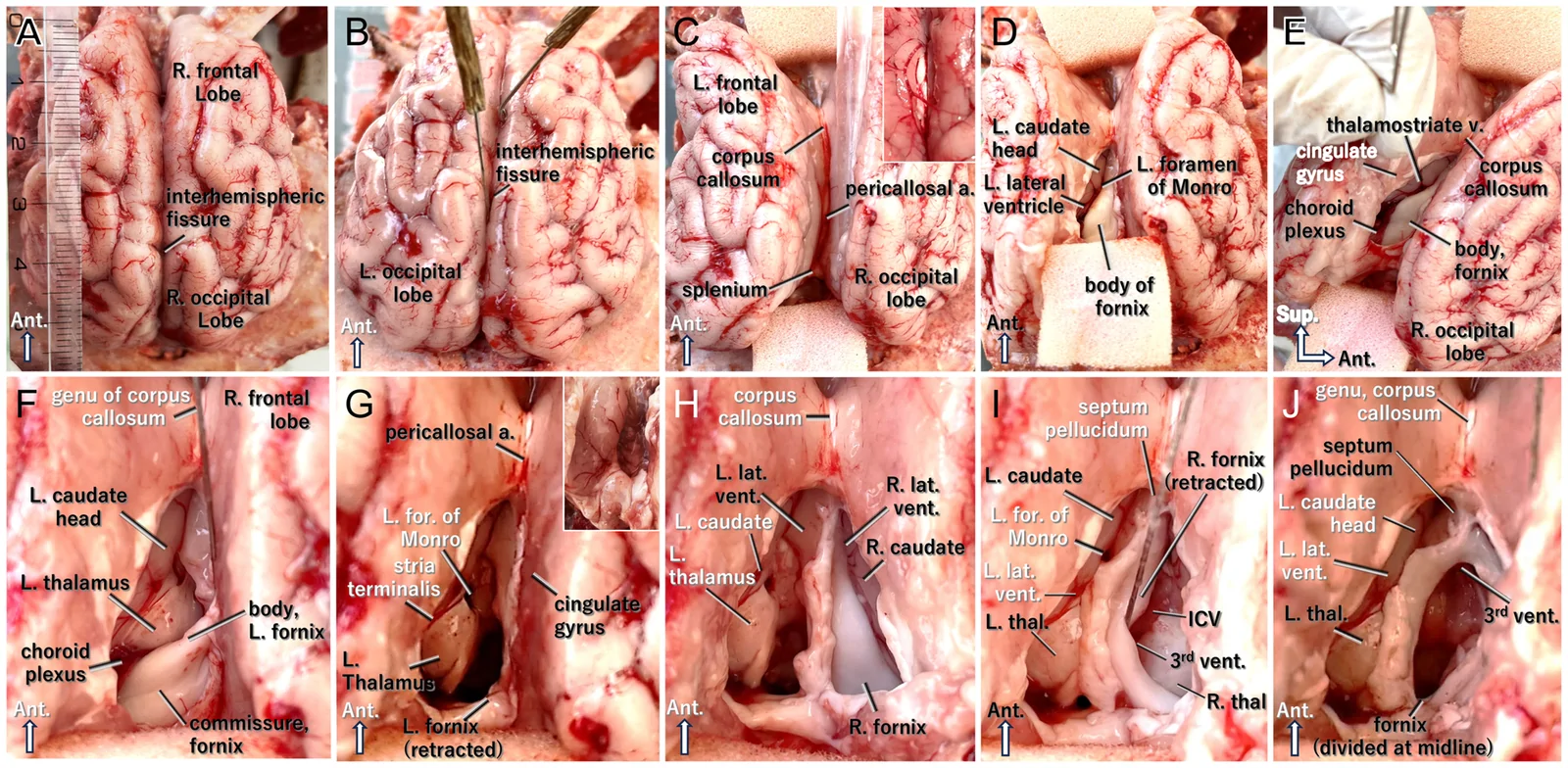
Stepwise interhemispheric, transcallosal, subchoroidal, and interforniceal dissection. A, Removal of the dura exposed the cerebral hemispheres. B, The interhemispheric fissure was opened using microsurgical technique, with preservation of bridging veins. C, The corpus callosum was identified as a pale commissural structure beneath the medial hemispheric surface; the inset shows the pericallosal arteries. D, A limited midline callosotomy permitted entry into the left lateral ventricle. E, The caudate nucleus, fornix, foramen of Monro, choroid plexus, and thalamostriate vein were visualized. F and G, Medial retraction of the fornix and choroid plexus exposed the thalamic surface and provided a subchoroidal trajectory toward the third ventricle. H and I, The contralateral ventricle was entered and dissected. J, Midline division of the fornix demonstrated the interforniceal route toward the third ventricle. A, artery; Ant, anterior; For, foramen; ICV, internal cerebral vein; L, left; Lat, lateral; R, right; Sup, superior; Thal, thalamus; V, vein; Vent, ventricle.

The thalamic surface and subchoroidal trajectory were demonstrated after mobilization of the fornix and choroid plexus (Fig 1F,G). The corresponding anatomy was also demonstrated in the contralateral ventricle (Fig 1H,I). The internal cerebral veins were identified ventral to the fornix, and the interforniceal view clarified the relationships among the fornix, thalami, internal cerebral veins, and roof of the third ventricle (Fig 1J and S2 Fig).

### Module 2: Transchoroidal exposure of intraventricular veins

The choroidal fissure, underlying thalamic surface, and small intraventricular venous tributaries were demonstrated in all five specimens (Fig 2A,B). The anterior caudate vein, posterior caudate vein, and anterior septal vein were directly visualized in all five specimens and identified on the basis of their observed courses and relationships with adjacent ventricular venous landmarks. The course of the thalamostriate vein into the velum interpositum and its continuity with the paired internal cerebral veins beneath the fornix were also demonstrated (Fig 2C).

**Fig 2:**
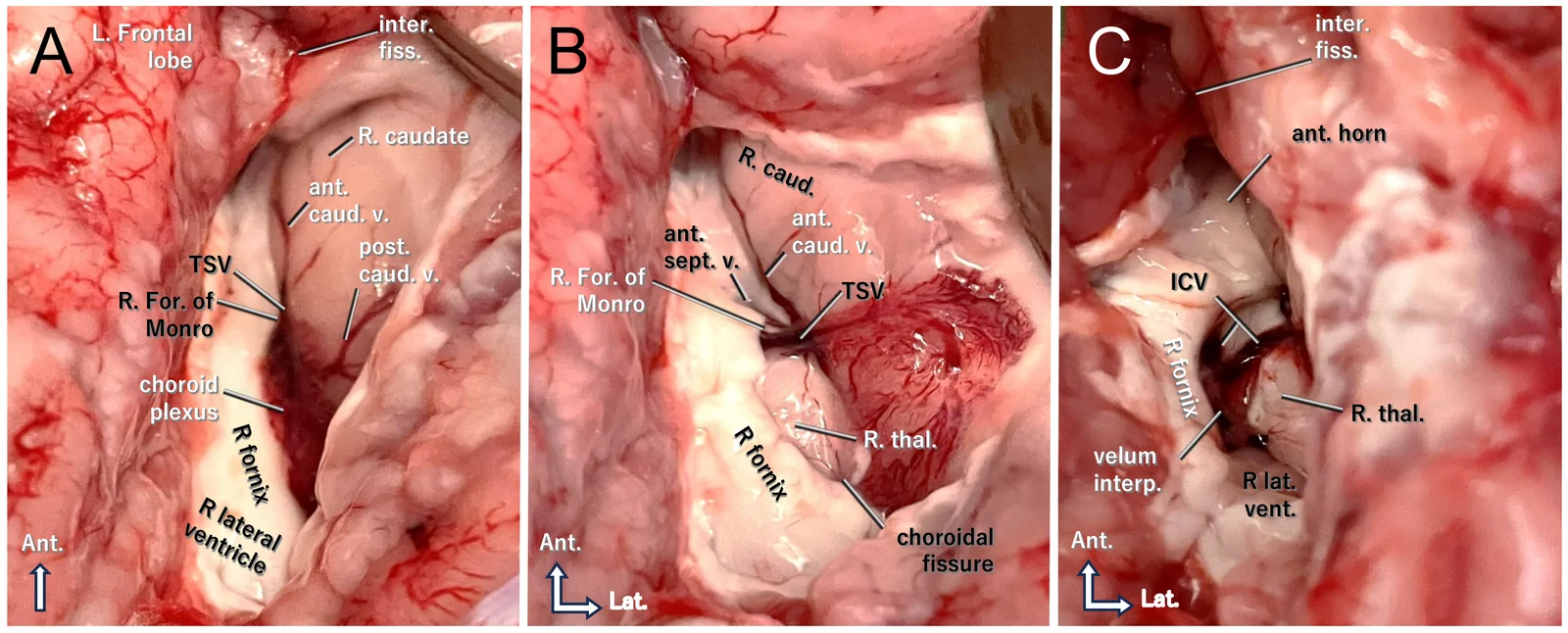
Transchoroidal identification of intraventricular veins. A, Transcallosal entry into the right lateral ventricle. B, The foramen of Monro was enlarged by opening the choroidal fissure and mobilizing the choroid plexus and thalamostriate vein laterally, exposing the thalamic surface. C, The thalamostriate vein was followed into the velum interpositum, where the paired internal cerebral veins were exposed. The anterior and posterior caudate veins are labeled in panel A, and the anterior caudate and anterior septal veins are labeled in panel B, showing their relationships to the thalamostriate vein. Ant, anterior; Caud, caudate; Fiss, fissure; For, foramen; ICV, internal cerebral vein; Inter, interhemispheric; Interp, interpositum; L, left; Lat, lateral; Post, posterior; R, right; Sept, septal; Thal, thalamus; TSV, thalamostriate vein; V, vein; Vent, ventricle.

### Module 3: Posterior continuity of the deep venous system

Posterior exposure demonstrated the relationship among the upper brainstem, tectal region, thalami, fornix, and deep cerebral veins in all five specimens (Fig 3A,B). The internal cerebral veins were traced posteriorly along the ventral surface of the fornix to the Galenic venous confluence (Fig 3C). Continuity from the vein of Galen to the straight sinus at the falcotentorial junction was also demonstrated (Fig 3D).

**Fig 3:**
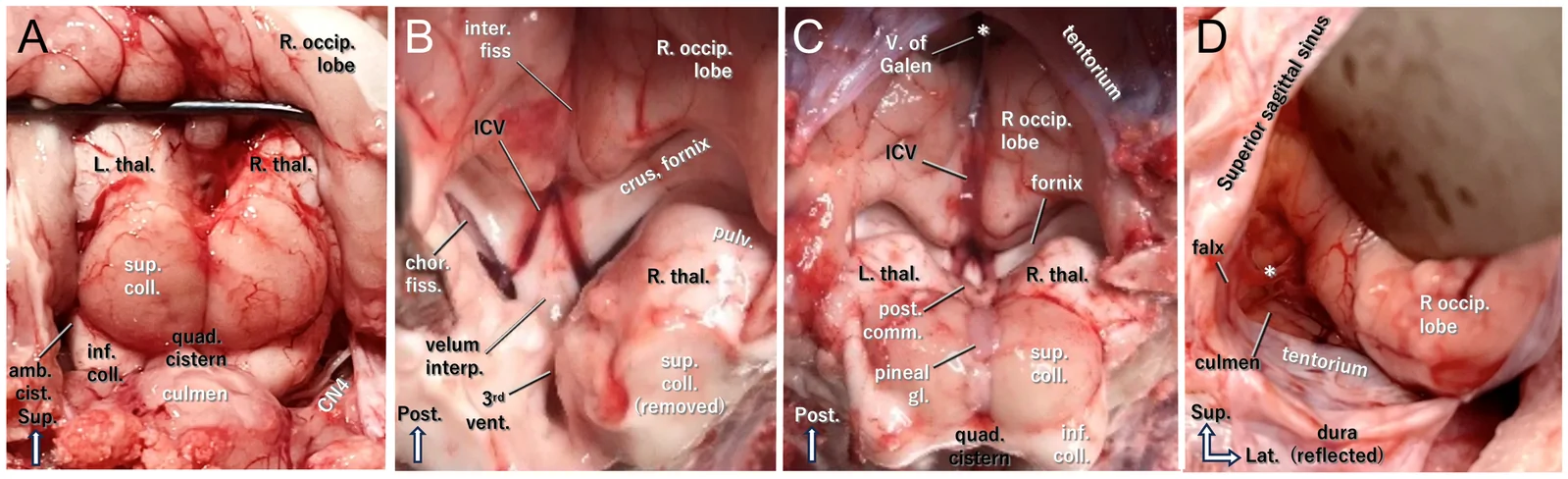
Posterior exploration of the deep venous drainage system. A, Posterior view of the upper brainstem and tectal region after superior retraction of the occipital lobes. B, Further retraction exposed the fornix and the internal cerebral veins on its ventral surface. C, The internal cerebral veins continued posteriorly toward the Galenic venous confluence, indicated by the white asterisk. D, At the falcotentorial junction, the vein of Galen drained into the straight sinus. Chor, choroidal; Cist, cistern; CN, cranial nerve; Coll, colliculus; Comm, commissure; Fiss, fissure; Gl, gland; ICV, internal cerebral vein; Inf, inferior; Inter, interhemispheric; Interp, interpositum; L, left; Lat, lateral; Occip, occipital; Post, posterior; Pulv, pulvinar; Quad, quadrigeminal; R, right; Sup, superior; Thal, thalamus; TSV, thalamostriate vein; Vent, ventricle.

### Module 4: Atrium, inferior horn, limbic structures, and adjacent cisterns

The atrium and inferior horn were exposed in all five specimens, providing a panoramic view of the posterior and inferior portions of the lateral ventricle (Fig 4A,B). The fornix and hippocampal formation were consistently observed as a continuous curved complex in relation to the thalamus (Fig 4A,C).

**Fig 4:**
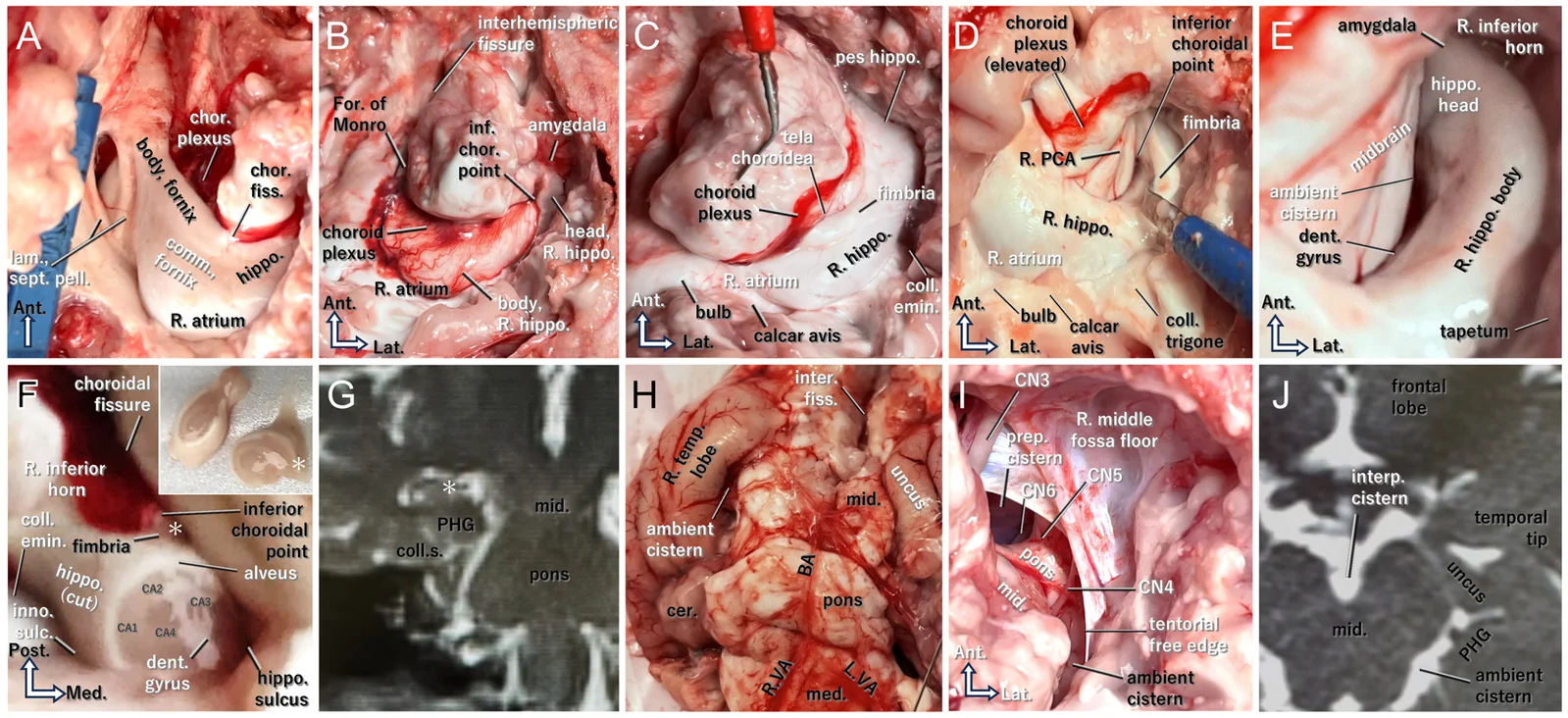
Atrium, inferior horn, limbic structures, and adjacent cisternal anatomy. A, Right atrium viewed from posterior, showing the curved continuity between the fornix and hippocampal formation in relation to the thalamus. B, The choroid plexus was followed along the choroidal fissure toward the inferior choroidal point. C, Medial mobilization of the choroid plexus exposed the fimbria. D, Opening the choroidal fissure provided access to the ambient cistern and posterior cerebral arterial branches. E, The hippocampal head was exposed within the inferior horn. F, Anterior view of the hippocampal head within the inferior horn; the inset shows sections through the hippocampal head, and the white asterisk indicates the fimbria. G, Coronal human T2-weighted magnetic resonance image at the hippocampal level for qualitative illustration. H, Whole porcine brain after removal from the cranium. I, Superior view of the right anterolateral brainstem and middle fossa after partial temporal lobe removal. J, Axial human T2-weighted magnetic resonance image at the midbrain level for qualitative illustration. A, artery; Ant, anterior; BA, basilar artery; CA, cornu ammonis; Cer, cerebellum; Chor, choroid or choroidal; CN, cranial nerve; Coll, collateral; Comm, commissure; Dent, dentate; Emin, eminence; For, foramen; Hippo, hippocampus; Inf, inferior; Inno, innominate; Interp, interpeduncular; L, left; Lat, lateral; Mid, midbrain; Occip, occipital; PCA, posterior cerebral artery; Pell, pellucidum; PHG, parahippocampal gyrus; Prep, prepontine; Quad, quadrigeminal; R, right; S, sulcus; Sept, septum; Temp, temporal; VA, vertebral artery.

The choroid plexus was traced to the inferior choroidal point, and the fimbria and choroidal fissure were demonstrated after medial mobilization of the choroid plexus (Fig 4B–D). Opening the choroidal fissure provided access to the ambient cistern, where posterior cerebral arterial branches were identified (Fig 4D). The hippocampal head and its relationship to adjacent mesial temporal structures were demonstrated within the inferior horn (Fig 4E,). Additional porcine anatomical views and human magnetic resonance images are presented for qualitative illustration only, without formal morphometric comparison (Fig 4G–J and S3 Fig).

## Discussion

### Principal findings

This study organized fresh porcine head dissection into a four-module sequence that connected ventricular entry, third ventricular routes, deep venous drainage, and the atrium-inferior horn continuum. The principal contribution is not the identification of a previously undescribed anatomical structure, but the integration of structures that are often learned separately into a single physical sequence. The preparation allowed the fornix, thalamus, choroidal fissure, internal cerebral veins, Galenic venous system, hippocampal formation, and adjacent cisterns to be followed as a continuous three-dimensional system.

### Relationship to previous large-animal models

Previous large-animal models have demonstrated the feasibility of practicing common cranial approaches, posterior fossa procedures, or general microsurgical techniques [1–4]. Our earlier porcine head study focused on introductory cranial approaches and gross anatomy [13]. In contrast, the present work uses a structured deep-anatomy sequence centered on ventricular-limbic and venous continuity. It is therefore conceptually closer to classical microsurgical anatomical studies and stepwise descriptions of approaches to the lateral and third ventricles [5–12], while using an accessible, non-fixed large-animal preparation.

### Porcine–human anatomical correspondence

Although the porcine preparation allowed consistent identification of many structures that have direct anatomical counterparts in the human brain, anatomical homology should not be interpreted as geometric equivalence [14–17]. The porcine and human brains differ in overall cerebral proportions, cortical organization, and the relative development and orientation of the temporal and olfactory regions [14,15,17]. These differences alter the spatial relationships and operative trajectories surrounding otherwise homologous deep structures. Table 2 therefore summarizes the principal qualitative correspondences and differences between the porcine structures demonstrated in this study and their human counterparts.

**Table 2:** Qualitative anatomical correspondence between porcine structures demonstrated in the present model and human neuroanatomy.

Porcine structure demonstrated	Qualitative correspondence to human anatomy	Important difference/caution
Corpus callosum and lateral ventricle	Major homologous landmarks are readily recognizable	Cerebral proportions and ventricular geometry differ
Foramen of Monro and third ventricular region	Comparable ventricular connections can be demonstrated	Relative dimensions and working angles differ
Choroid plexus and choroidal fissure	Fundamental anatomical relationships are recognizable	Exact orientation and dimensions differ
Thalamus	Serves as a central deep landmark in both species	Relative proportions and spatial relationships to surrounding hemispheric structures differ
Fornix–hippocampal complex	Hippocampal organization shows substantial structural correspondence with human anatomy	Exact geometry of the fornix–hippocampal complex was not formally compared between species in the present study
Internal cerebral veins and Galenic venous system	Major components of the deep venous system are broadly comparable and recognizable	Correspondence of individual venous tributaries with human anatomy was not formally validated, and relative venous geometry may differ
Atrium and inferior horn	Corresponding ventricular regions can be exposed	Overall cerebral proportions and orientation differ from humans
Ambient/quadrigeminal cisternal regions	Deep cisternal relationships can be demonstrated	Cranial and brainstem geometry, dimensions, and operative angles differ

Note. Porcine anatomical relationships are based on observations from the present dissections together with established porcine neuroanatomical atlases and comparative literature [14–18]; human anatomical relationships are based on established microsurgical anatomical literature [5–12]. The comparisons are qualitative, and no formal morphometric comparison between species was performed.

The ventricular system, thalamus, choroid plexus, choroidal fissure, and major components of the deep venous system provide recognizable relational landmarks in the porcine specimen. In contrast, greater caution is required when interpreting the fornix–hippocampal complex and mesial temporal anatomy. Although a continuous curved relationship between the fornix and hippocampal formation was consistently demonstrated in the present specimens, its geometry should not be assumed to reproduce the human C-shaped configuration precisely. Accordingly, the model is best regarded as a physical reference for selected three-dimensional relationships and dissection planes rather than as a geometrically faithful representation of human operative anatomy.

### Anatomical and potential educational value

Deep ventricular anatomy is frequently taught as a collection of separate landmarks. In operative reality, orientation changes as the surgeon moves from the lateral ventricle toward the third ventricular roof, the velum interpositum, the deep venous confluence, or the inferior horn. The present sequence emphasizes these transitions. Transcallosal entry establishes intraventricular orientation; transchoroidal and interforniceal dissections demonstrate access around the roof of the third ventricle; posterior dissection follows deep venous drainage; and inferolateral extension demonstrates the continuous relationship among the fornix, hippocampal formation, choroidal fissure, and thalamus.

A physical model may complement textbooks, clinical imaging, digital models, and operative videos by allowing learners to manipulate tissue planes and repeatedly reorient the specimen. However, the present study did not evaluate knowledge acquisition, technical performance, skill retention, or transfer to human surgery. These educational outcomes require prospective assessment before the model can be described as an effective training intervention.

### Practical advantages and boundaries

The fresh porcine preparation has several practical advantages. It does not require formalin fixation, vascular injection, or dedicated cadaver laboratory infrastructure. The compact brain permits simultaneous observation of local microsurgical anatomy and gross cerebral topography. Fresh tissue retains malleability and recognizable tissue planes, and naturally colored vessels provide visual landmarks without injection. These features may be useful where access to injected human specimens or advanced simulation facilities is limited.

These anatomical boundaries, summarized in Table 2, should be considered when using the model. The preparation should therefore not be presented as a replica of human operative anatomy. Its appropriate role is as an adjunctive physical reference for selected relational concepts, used together with human imaging, validated anatomical resources, supervised laboratory education, and, where available, human cadaveric dissection.

### Limitations

This study has several limitations. First, only five specimens were used, limiting evaluation of anatomical variation and the effects of age, breed, sex, and postmortem interval. Second, all dissections were performed by a single team at one institution. Although an independent blinded image-based assessment was performed, it was limited to three specimens with complete unannotated photographic sets and a single assessor; inter-rater reliability was not evaluated. Third, no formal morphometric comparison was performed with human cadaveric specimens or imaging datasets; porcine-human similarities are therefore qualitative and relational. Accordingly, the anatomical correspondences summarized in Table 2 should be interpreted qualitatively and should not be taken as evidence of geometric equivalence between porcine and human anatomy. Fourth, learner outcomes and surgical skill acquisition were not assessed. Fifth, the independent image-based assessment was limited by the quality and timing of the available photographs. In some specimens, cerebrospinal fluid drainage and the resulting loss of ventricular distension may have reduced the separation between intraventricular structures, making small veins and ventricular landmarks more difficult to identify retrospectively on static images. This may partly explain why several targets that were identified during direct dissection were not consistently identifiable across the three evaluable photographic sets. Smartphone photography and the absence of vascular injection may also have limited visualization of fine vessels. Finally, tissue acquisition, biosafety, and disposal requirements differ among institutions and jurisdictions.

## Conclusions

Fresh porcine heads can be organized into a stepwise *ex vivo* model for demonstrating selected ventricular, limbic, choroidal fissure, cisternal, and deep venous relationships. The model provides a low-infrastructure physical reference for following the three-dimensional continuity of structures surrounding the thalamus and upper brainstem. It should be considered an adjunct to, rather than a substitute for, human anatomical dissection, clinical imaging, and supervised neurosurgical training. Prospective learner studies and formal anatomical comparison are needed to establish its educational effectiveness and define the limits of transfer to human surgery.

## Supporting information

S1 Fig Setup for porcine craniotomy and dissection. A and B, A manual saw and simple improvised microdissection instruments were used. C, Lateral view of a fresh porcine head after removal of the skin, mandible, and oropharynx. D, Superior view after detachment of the temporalis muscle and zygomatic arches. E, The transverse craniotomy created a stable box-like preparation for tabletop dissection. F, Removal of the calvarium and dura exposed the cerebral hemispheres. Ant, anterior; Orb, orbital; R, right; Sup, superior; Temp, temporal.
(TIFF)

S2 Fig Spatial organization of the fornix, thalamus, and deep cerebral veins. A, Axial section showing the flat triangular fornix between the caudate nuclei. B, Medial retraction of the fornix exposed the thalamic surface. C, Further retraction exposed the internal cerebral veins. D, Reflection of the fornix exposed the thalami and roof of the third ventricle. E, Posterior reflection exposed the quadrigeminal cistern and deep cerebral veins. A, artery; Ant, anterior; Chor, choroidal; Coll, colliculus; Fiss, fissure; For, foramen; ICV, internal cerebral vein; Inf, inferior; L, left; Lat, lateral; Quad, quadrigeminal; R, right; Sup, superior; Termin, terminalis; Thal, thalamus; TSV, thalamostriate vein; V, vein; Vent, ventricle.
(TIFF)

S3 Fig Qualitative comparison of porcine and human midline brain anatomy. The black asterisk indicates the thalamus. A, Midline sagittal section of the fresh porcine brain in situ. B, Midline sagittal human T1-weighted magnetic resonance image. C, Midline sagittal section of the porcine brain after removal from the cranium. D, Midline sagittal human T2-weighted magnetic resonance image. The images illustrate broad qualitative relationships but are not a morphometric comparison. Ant, anterior; Caud, caudal; Cereb, cerebellum; For, foramen; ICV, internal cerebral vein; N, nerve; Sup, superior; Thal, thalamus; TSV, thalamostriate vein.
(TIFF)

S1 Data Specimen-level identification of anatomical targets. The dataset provides identification results for each prespecified anatomical target in all five porcine specimens, together with independent image-based assessment results for the three evaluable specimens.
(XLSX)
